# Oxygen shortage: Himalayan adventures in an incubator

**DOI:** 10.1038/s44318-024-00105-5

**Published:** 2024-05-02

**Authors:** Nathalie M Mazure

**Affiliations:** 1 DR1 CNRS, Nice, France; 2 Université Côte d’Azur, INSERM U1065, Centre Méditerranéen de Médecine Moléculaire, Nice, France; 3 Equipe labellisée La Ligue contre le Cancer, Nice, France

**Keywords:** Metabolism, Methods & Resources

## Abstract

Recent work uncovers local hypoxia in standard cell culture due to excessive cellular oxygen consumption, demanding careful control of cell density and medium volume.

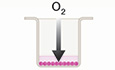

## Teaser

Every day, cells face hypoxic conditions without anyone realizing it. New research by Tan et al (2024) reveals that differentiated cells in standard cell culture medium volumes experience local hypoxia, with significant implications for cell metabolism and beyond, demanding a careful understanding and control of oxygen levels in in vitro experiments to better translate them in vivo.

Hypoxia, or reduced oxygen availability, is a fundamental physiological stress that cells encounter in various microenvironments, both in health and disease. To cope with hypoxic conditions, cells activate a sophisticated adaptive response orchestrated by a family of transcription factors known as Hypoxia-Inducible Factors (HIFs) (Brahimi-Horn et al, 2007; Brahimi-Horn and Pouyssegur, 2009; Schito and Semenza, 2016). HIFs play a pivotal role in mediating cellular responses to hypoxia by regulating the expression of genes involved in a wide range of biological processes, including angiogenesis, glucose metabolism, cell proliferation, and apoptosis. Through their ability to modulate gene expression in response to changes in oxygen tension, HIFs enable cells to maintain homeostasis and survive under low oxygen conditions. Dysregulation of HIF-mediated responses to hypoxia is associated with numerous diseases, including cancer, ischemic disorders, and chronic inflammatory conditions. Thus, understanding the molecular mechanisms underlying HIF activation and its downstream effects on cellular function is of great interest in biomedical research.

Comparing normoxia and hypoxia in tissues and cell culture involves assessing cellular and physiological responses under controlled oxygen conditions. Normoxia, representing normal oxygen levels similar to ambient air, contrasts with hypoxia, where oxygen levels are reduced, typically ranging from 1 to 5% depending on experimental setup. To conduct comparisons, precise oxygen control systems are utilized in incubators or chambers to maintain desired oxygen levels consistently. Cellular responses to normoxia and hypoxia are evaluated through assays measuring viability, proliferation, apoptosis, and metabolic activity, along with gene expression and signaling pathway analyses. Physiological responses may include oxygen consumption, blood oxygen saturation, tissue perfusion, and systemic adaptations like erythropoiesis and angiogenesis. Functional studies shed light on cellular metabolic shifts and adaptive responses mediated by HIFs. Through rigorous data analysis, differences between normoxia and hypoxia responses are identified, facilitating a deeper understanding of how oxygen levels influence cellular behavior and adaptation mechanisms across various biological contexts.

**Figure 1:**
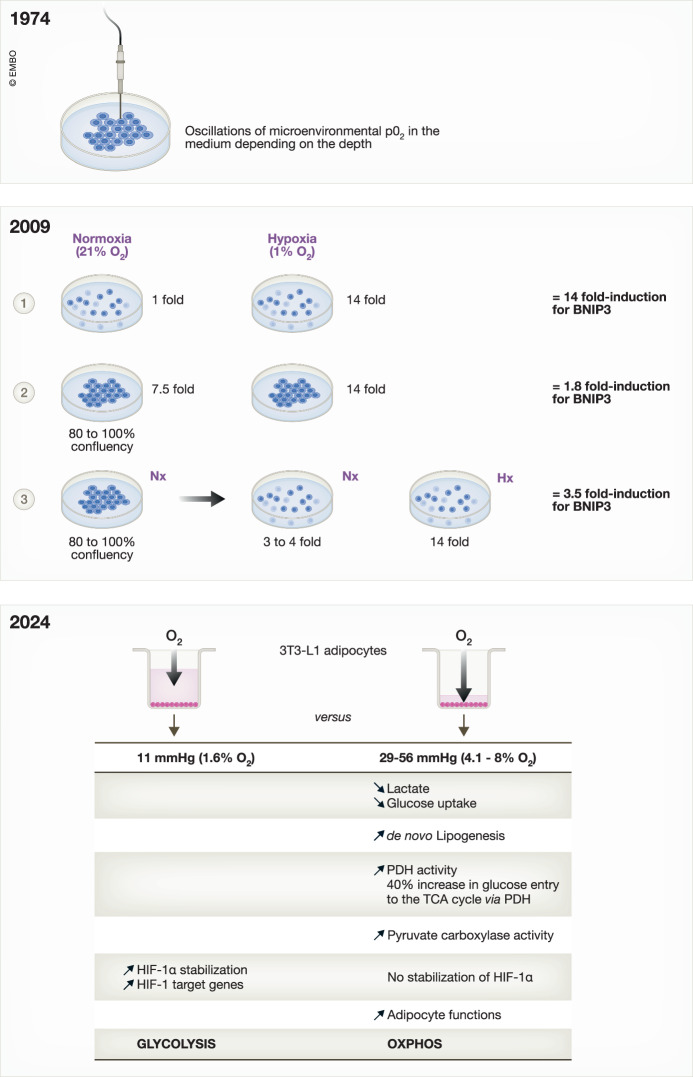
Temporal progression in assessing the impact of cell density and medium volume in cell culture. **1974**—Utilization of micro-oxygen cathode to measure O_2_ levels based on depth of medium volume (Werrlein and Glinos, 1974). **2009**—Expression of BNIP3 analyzed in normoxic and hypoxic conditions across various cell confluence levels (Dayan et al, 2009). **2024**—Lowering medium induces metabolic alterations (Tan et al, 2024).

If the conditions of hypoxia are adequately ensured, what about the conditions of normoxia? Already in 1974, Werrlein and Glinos proposed a groundbreaking idea, suggesting that the oxygen transport within cultures of attached cells relies on the kinetics of gas diffusion through the overlaying medium (Werrlein and Glinos, 1974). Utilizing micro-oxygen cathodes, they detected oscillating concentration gradients of oxygen above the cell layer, ranging from 20% O_2_ at 500 µm to 2.8% O_2_ at cellular level (Fig. 1—1974). Remarkably, the oxygen tension exhibited a marked decline in a directly inverse relationship to increasing density. Fast forward 25 years, Tokuda et al (2000), using a dissolved oxygen probe, described a linear decrease in oxygen tension with increased cell density in both human dermal fibroblasts and NIH/3T3 cultures. Just one year later, Sheta et al (2001) demonstrated that under routine normoxic culture conditions, cell density leads to pericellular hypoxia, mediating the stabilization of HIF-1α protein and subsequent increased HRE activity. Moreover, they proposed that Ras and MEK1 mediated the activity of HRE in response to nitric oxide as a diffusible factor secreted by densely cultured cells in standard culture conditions. In 2009, while studying the stabilization kinetics of HIF-1α under hypoxia for 24, 48, and 72 h, we observed HIF-1α stabilization under control conditions in normoxia (Dayan et al, 2009). However, the kinetics of HIF-1α in normoxia were unstable and non-reproducible. The only thing that was truly stable in normoxia was the induction of HIF-1 target genes. We therefore decided to question our normoxia conditions and showed that the accumulation of HIF-1α during normoxia at high cell density is attributed to increased oxygen consumption by cells as supported using a respiration inhibitor, oligomycin, and respiratory-defective mutant cells (GSK3). Furthermore, our study revealed a decrease in AKT activity and p70S6K phosphorylation, indicating reduced mTOR activity during high oxygen consumption caused by high cell density. In contrast, hypoxia had minimal impact on the mTOR pathway under low cell density conditions. We concluded that HIF-1α activation in exponentially growing cells via hypoxic stimulation was independent of the Akt/mTOR pathway. However, HIF-1α activation, estimated at 3% O_2_ due to high cell density conditions, relied on the mTOR pathway.

From these findings, it becomes clear how misunderstandings regarding cell culture and confluence can lead to numerous pitfalls. Consider, for instance, the straightforward case of BNIP3 expression for imaging (Fig. 1—2009). In the first case, we compare a low-confluence cell culture exposed to normoxic conditions in a cell culture incubator to a low-confluence cell culture exposed to hypoxic conditions. BNIP3, a target gene of HIF-1, will then be expressed ~14 times under hypoxia and compared to a value of 1 under normoxia. This yields a fold induction of 14. In the second case, we still compare our culture under hypoxia with a BNIP3 expression induction of 14 to a normoxic cell culture where cells are in abundance with high confluence for at least 24 to 48 h. BNIP3 expression will already be at 7.5-fold induction, but since this expression is your control, you bring it down to 1, resulting in a BNIP3 induction under hypoxia of 1.8. In the third case, you let your cells grow all weekend, your plate is full on Monday, but you read somewhere that confluence can lead to hypoxia, so you decide to pass your cells and conduct your experiment on Tuesday. Certainly, HIF-1α will have disappeared, but this does not consider the half-life of BNIP3. In normoxia, its expression has decreased and is between 3 and 4-fold induction, which you will compare to your value of 14 under hypoxia. BNIP3 is then induced by a factor of 3.5. Reproducing a 14-fold induction of BNIP3 thus requires rigor, which we, and others, have maintained since then.

In the domain of hypoxia research, ongoing inquiries persist regarding the optimal oxygen concentration to utilize, the physiological oxygen concentration within the body (which will be discussed later), the most effective oxygen partial pressure, and so forth. Addressing these concerns, Wenger et al (2015) provided a comprehensive examination of these questions including the inquiry into the oxygen concentration in a normoxic incubator. Utilizing Dalton’s law, they calculated that the “true” normoxic oxygen condition in everyday cell culture should be around 18.6% O_2_ only at sea level. However, oxygen concentration should increase with increasing altitude, adding another layer of complexity to normoxic culture practices. Furthermore, the mechanism by which O_2_ reaches the bottom of the tissue culture dish or flask is primarily diffusion, which is often the limiting factor for cellular oxygenation. Another fundamental law, Fick’s law, establishes that diffusion is directly proportional to the partial pressure, solubility, and inversely proportional to the diffusion distance. Based on this principle, O_2_ diffusion has been estimated to reach approximately 100 to 200 µm. Consequently, it is easy to understand that in a Petri dish, this inevitably results in a low pericellular pO_2_ and poor cellular oxygenation, leading to hypoxic conditions even under normoxic incubator settings.

Allow me to emphasize a practical observation from Wenger. “So, how can this problem be solved? The usual approach is to ignore it and to simply compare “normoxic” with “hypoxic” exposure under otherwise identical conditions, knowing that these expressions refer to the incubator’s air composition only and have nothing to do with the physiological tissue situation.” Applying this theory, a deluge of articles has emerged featuring cultures spanning several days, genes left uninduced under hypoxia due to inadequate controls, and, most notably, results obtained under hypoxic conditions but deceptively presented as if they were garnered under normoxia. In our instance, we chose the cell culture marked as #1 in Fig. 1, acknowledging the challenges presented by hypoxic conditions even within normoxia.

Since 1974, techniques have evolved, and the use of oxygen probes has become obsolete to emphasize the importance of controlling normoxic conditions. A new study by Tan et al (2024) demonstrated that terminally differentiated cells experience a degree of hypoxia under standard culture conditions named normoxia. Tan and colleagues (2024) present compelling evidence through a series of biochemical, multi-omics, and phenotypic analyses, that various cell types in culture encounter local hypoxia, which has significant implications for cell metabolism and, crucially, cell function (Fig. 1—2024). Initially focusing on 3T3-L1 adipocytes, given the frequent observation of adipose tissue hypoxia in obesity preceding diminished adipocyte function, they discovered that cultured adipocytes under standard conditions exhibit highly glycolytic activity and a transcriptional profile indicative of physiological hypoxia. Increasing pericellular oxygen by reducing medium volumes elevated pericellular oxygen levels, redirected glucose flux towards mitochondria, decreased HIF-1 activity, and led to widespread transcriptional rewiring. Furthermore, they demonstrated that lactate production correlated with cell density and oxygen consumption, underscoring how cell density can influence diverse cellular metabolic profiles. Reducing medium volumes also influenced lactate production in cardiac organoids. Certainly, Tan et al (2024) not only addressed the significance of cell density and medium volume but also explored the diverse dynamics of cell types with varying proliferation rates, encompassing both cancerous and immune cells. In this article, Tan and team (2024) also briefly address another interesting aspect: the relevance to culturing our cells under hyperoxic conditions, at 21% O_2_. They suggest that the optimal oxygen tension for a specific cell type would ideally correspond with the oxygen levels present in the relevant tissue in vivo, serving as the most suitable reference point. Indeed, very few of our cells in the human body are subjected to such oxygen concentrations. At sea level, the oxygen partial pressure (PO_2_) of inspired air typically measures around 21% O_2_ (Brahimi-Horn et al, 2007). Nevertheless, a significant decrease in PO_2_ occurs in the lungs, partially due to water vapor and diffusion. As blood flows from the alveolar capillaries, it carries oxygen at a PO_2_ of approximately 14% O_2_ to organs and tissues for oxygenation. The normal PO_2_ of specific tissues varies; for instance, the rat spleen has a measured PO_2_ of around 2.2% O_2_, while for the thymus, it is ~1.4% O_2_ (Braun et al, 2001). Even the normal rat retina, due to its low vascularity, exhibits relative hypoxia ranging approximately from 0.3 to 3.5% O_2_ (Yu and Cringle, 2005). The normal tissue of the rat brain is even more hypoxic, ranging from 0.05 to 1.1% O_2_, depending on the location (Erecinska and Silver, 2001). Consequently, certain normal tissues can be considered hypoxic. However, we prefer to refer them as *physioxic*. At one point, we considered the possibility of adapting our cells to 6% O_2_ levels when during our investigation of medulloblastoma (Contenti et al, 2023), or 4% O_2_ level when studying prostate cancer cells, maintaining consistently culture at these percentages. In that regard, we fully support the findings and conclusions presented by Tan et al (2024).

Finally, it’s important to note that all these O_2_% have been obtained at sea level. While the percentage of oxygen in inspired air remains consistent across different altitudes, the decrease in atmospheric pressure at higher altitudes diminishes the partial pressure of inspired oxygen, thereby reducing the driving pressure for gas exchange in the lungs. Consequently, oxygen distribution will vary accordingly. Therefore, experiments and results conducted in locations like Cambridge or Nice may yield different results compared to those conducted in Denver, owing to variations in altitude.

Based on all this accumulated knowledge, and despite 40 years of research, data, and explanations summarized here, scientists still persist in working in normoxia at 21% without considering crucial factors such as cell density, volume, and gas diffusion. Let us hope that Tan et al (2024) will succeed in reaching the younger generation of researchers and bring about a necessary shift in our approach.
